# Potential Mechanisms of Epicardial Adipose Tissue Influencing Heart Failure with Preserved Ejection Fraction

**DOI:** 10.31083/j.rcm2509311

**Published:** 2024-09-03

**Authors:** Qiuxuan Li, Ur Rehman Muhib, Xiaoteng Ma, Zaiqiang Liu, Fei Gao, Zhijian Wang

**Affiliations:** ^1^Department of Cardiology, Beijing Anzhen Hospital, Capital Medical University, Beijing Institute of Heart Lung and Blood Vessel Disease, Beijing Key Laboratory of Precision Medicine of Coronary Atherosclerotic Disease, Clinical Center for Coronary Heart Disease, 100029 Beijing, China

**Keywords:** HF, HFpEF, epicardial adipose tissue, visceral adipose tissue

## Abstract

Heart failure (HF) is the predominant terminal stage and the leading cause of mortality in cardiac disease. Heart failure with preserved ejection fraction (HFpEF) affects roughly 50% of HF patients globally. Due to the global aging population, the prevalence, morbidity, and mortality of HFpEF have gradually increased. Epicardial adipose tissue (EAT), as a key visceral adipose tissue around the heart, affects cardiac diastolic function and exercise reserve capacity. EAT closely adheres to the myocardium and can produce inflammatory factors, neurotransmitters, and other factors through autocrine or paracrine mechanisms, affecting the heart function by inflammatory response, cardiac metabolism and energy supply, cardiomyocyte structure and electrical activity, and pericardial vascular function. Currently, research on the mechanism and treatment methods of HFpEF is constantly improving. EAT may play a multi-level impact on the occurrence and development of HFpEF. This review also summarizes the potential impact of EAT on the heart in HFpEF combined with other metabolism-related diseases such as obesity or diabetes over other obesity-related measures, such as body mass index (BMI) or other adipose tissue. Above all, this review comprehensively summarizes the potential mechanisms by which EAT may affect HFpEF. The objective is to enhance our comprehension and management of HFpEF. Future research should delve into the mechanistic relationship between EAT and HFpEF, and investigate interventions aimed at EAT to improve the prognosis of patients with HFpEF.

## 1. Introduction

Heart failure (HF) is the predominant terminal stage and the leading cause of mortality in cardiac disease. Various conditions, including coronary artery disease (CAD), rheumatic heart disease, hypertensive heart disease, and chronic pulmonary heart disease frequently advance to HF. Epidemiology has shown that heart failure with preserved ejection fraction (HFpEF) affects approximately 50% of HF patients globally, with its prevalence, morbidity and mortality rates progressively rising [1]. Due to the global aging population, HFpEF has garnered increasing academic interest. There is a positive correlation between the incidence of HFpEF and advancing age, whereby the severity of HFpEF becomes more pronounced with age than heart failure with reduced ejection fraction (HFrEF), and the prevalence of HFpEF is higher in women than in men across all age groups [1].

Epicardial adipose tissue (EAT) is predominantly situated in the atrioventricular and interventricular grooves of the heart, encompassing roughly 80% of the cardiac surface [2]. EAT closely adheres to the myocardium. Owing to its non-fascial composition, it can affect cardiac function via autocrine or paracrine mechanisms. Abnormal accumulation or alteration of EAT can influence the onset and progression of HFpEF, surpassing the significance of body mass index (BMI) or central obesity [3, 4]. The study examined the association between EAT and the all-cause mortality rate as well as the hospitalization rate in HFpEF patients. The findings indicated a positive correlation between these clinical outcomes and EAT accumulation, while no significant association with BMI accumulation [5]. Consequently, this review aims to provide a comprehensive overview of the underlying mechanisms between EAT and the occurrence and progression of HFpEF.

## 2. Heart Failure with Preserved Ejection Fraction 

HF is characterized as a clinical syndrome caused by impaired cardiac ejection function, which subsequently leads to hemodynamic instability. This condition is typically accompanied by pertinent clinical symptoms and signs. According to the classification outlined in the 2016 European Heart Failure Guidelines [6], HF can be categorized into three groups based on left ventricular ejection fraction (LVEF): HFrEF when LVEF is less than 40%, heart failure with mid-range ejection fraction (HFmrEF) when LVEF is between 40% and 49%, and HFpEF when LVEF is 50% or greater. In a multinational multicenter prospective longitudinal study [7], HFpEF was found to have a two-year mortality rate of 14%, while the composite endpoint of all-cause mortality rate or HF hospitalization rate was 35%. These findings highlight the significant healthcare burden imposed on society by HFpEF.

In the past, researchers considered HFpEF solely a clinical syndrome related with diastolic dysfunction. However, in recent years researchers have defined it as a systemic clinical syndrome that affects multiple organs, including the heart, lungs, kidneys, and skeletal muscles [8]. HFpEF is also characterized by a high prevalence of non-cardiac complications. Compared to HFrEF and HFmrEF, HFpEF is associated with a greater risk of comorbidity, resulting in higher rates of all-cause readmissions and non-cardiovascular mortality [9, 10]. The main clinical phenotypes and independent risk factors of HFpEF include obesity [11, 12, 13], hypertension [14], atrial fibrillation (AF) [15], and type 2 diabetes mellitus (T2DM) [16]. HFpEF is diagnosed as a syndrome through the “HFA-PEFF diagnostic algorithm” (include four steps: pre-test assessment, echocardiography and natriuretic peptide score, functional testing, and final aetiology) proposed by the European Society of Cardiology [17] and the “H2FPEF scoring system” (a composite score ranging from 0–9) proposed by Reddy *et al*. [18]. The diagnosis is based on comprehensive evaluation of clinical symptoms, signs, auxiliary examinations, and laboratory tests. Multiple mechanisms contribute to the occurrence and development of HFpEF, including significant factors such as ventricular diastolic dysfunction [19, 20], microvascular dysfunction [21, 22], inflammation [23], fibrosis [24, 25], nitric oxide dysfunction [16, 26], and insulin resistance [27, 28]. In a study published in the European Heart Journal in 2018, Lam *et al*. [29] systematically summarized three hemodynamic mechanisms and three molecular mechanisms of HFpEF from macroscopic cardiac function to microscopic molecular alterations.

## 3. Epicardial Adipose Tissue

EAT is visceral fat located between the myocardial surface and the visceral epicardium and plays certain physiological roles [30, 31]: (1) EAT serves as a protective layer for the coronary arteries and myocardium, providing mechanical buffering and potentially exerting localized pressure on the myocardium; (2) EAT is more like brown adipose tissue (BAT) and contains a large number of uncoupling protein-1 (UCP-1), thereby facilitating the maintenance of normal energy metabolism in cardiac tissues; (3) EAT can release adipokines or cytokines to regulate cardiac metabolic activity within normal levels; (4) EAT is abundant in saturated fatty acids (FAs) and can absorb or metabolize free FAs to maintain cardiac FAs balance. Non-invasive imaging modalities can be used to quantify EAT. Magnetic resonance imaging (MRI) is considered the gold standard for measurement in clinical [32]. Additionally, echocardiography as a more cost-effective and convenient method, can also be utilized for the measurement and assessment of EAT [33].

**Fig. 1.**
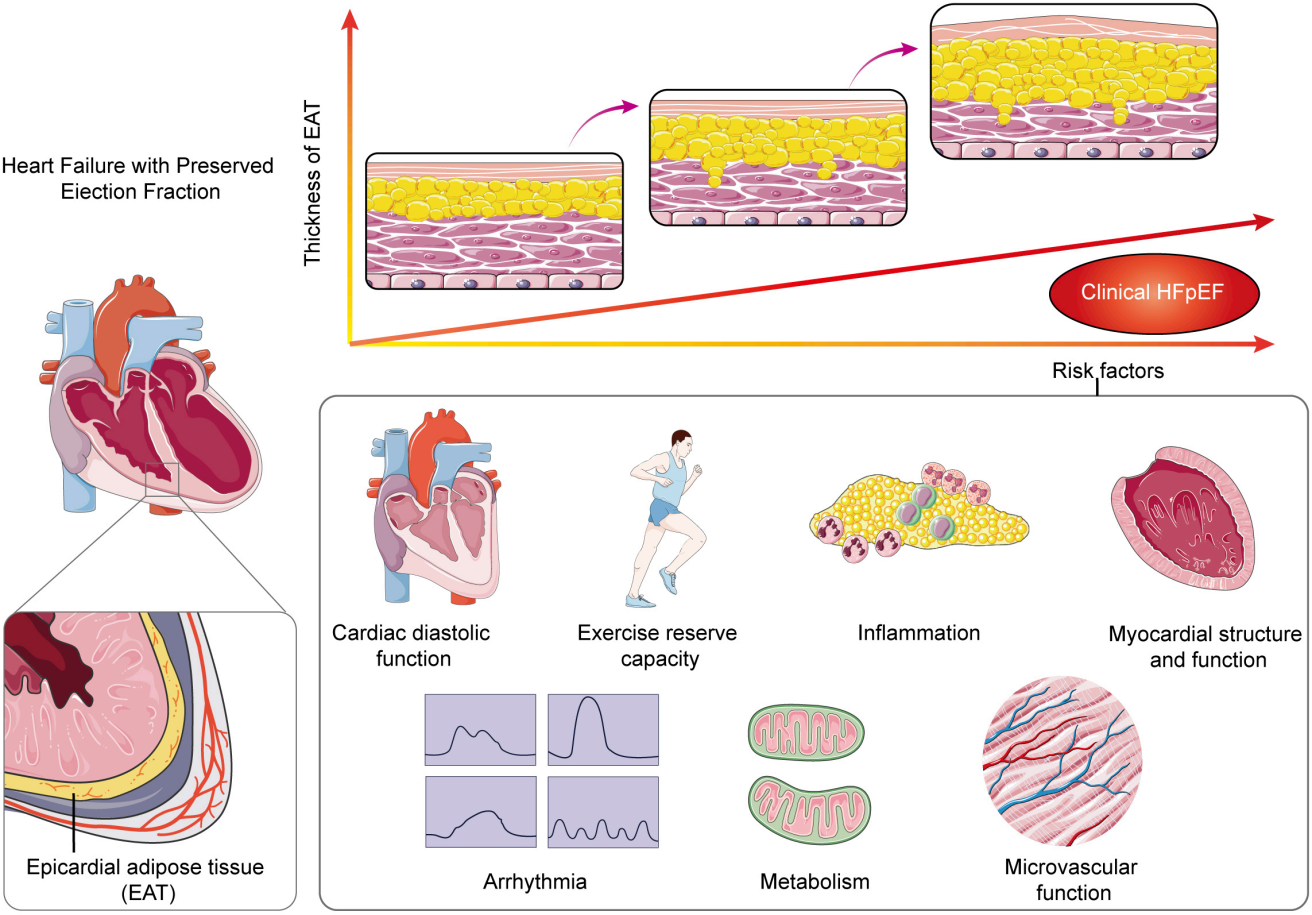
**Schematic overview of the pathways by which epicardial adipose tissue (EAT) may accumulate and eventually affect the underlying myocardium leading to left ventricular (LV) hypertrophy, LV diastolic dysfunction**. HFpEF, heart failure with preserved ejection fraction.

According to clinical studies, there are differences in the accumulation and impact of EAT between patients with HFpEF or HFrEF/HFmrEF. In comparison to HFrEF/HFmrEF patients, HFpEF patients tend to exhibit greater accumulation of EAT, and the size and thickness of EAT are positively correlated with the degree of atrial and ventricular functional impairment [34]. Conversely, HFrEF/HFmrEF patients with larger EAT mass are associated with greater cardiac function [35, 36]. Therefore, the role of EAT in different types of HF is not solely determined by volume or mass. Numerous studies have demonstrated a positive correlation between atrial or ventricular dysfunction and EAT volume in HFpEF patients [36], but no direct correlation has been observed with the distribution location of EAT [37, 38]. These findings strongly support that EAT impacts myocardial function primarily through paracrine mechanisms rather than direct infiltration or physical interaction with the myocardium. EAT exhibits a significant correlation with various diseases including AF [39, 40], T2DM [41], obesity [42], and hypertension [43], all of which frequently coexist with HFpEF. Individuals with elevated EAT volume exhibit inflammation, oxidative stress, and endothelial dysfunction, along with blood glucose and lipid metabolism disorder, all of which constitute critical elements of HFpEF pathophysiology [44]. Classified as a unique form of visceral adipose tissue (VAT), EAT demonstrates a close association with obesity [45]. In the “PROMIS-HFpEF” (Prevalence of Microvascular Dysfunction in HFpEF) cohort study conducted across various countries, the influence of EAT on HFpEF may be modulated by obesity [44]. Nevertheless, in a prospective multicenter study [5], it was observed that HFpEF patients with obesity exhibited a notably higher occurrence of adverse events in individuals with substantial EAT accumulation than in those with minimal EAT accumulation. This finding implies that EAT might exert an autonomous effect on HFpEF (Fig. 1).

## 4. Mechanisms of EAT Influencing HFpEF 

### 4.1 Cardiac Diastolic Function

HFpEF was defined as HF with impaired diastolic function. Due to ongoing studies on HF and its classification, the definition of HFpEF has been corrected. However, diastolic dysfunction remains to be recognized as a significant pathological mechanism of HFpEF. A comprehensive meta-analysis encompassing 21 studies has demonstrated that EAT plays a significant role in the development of cardiac diastolic dysfunction [46]. Although EAT exhibits a correlation with obesity, and obesity is independently associated with diastolic dysfunction, EAT possesses a superior predictive capacity for cardiac diastolic function compared to BMI and other relevant indicators, particularly in patients with T2DM [47]. Echocardiography results showed that patients with HFpEF who had elevated EAT exhibited a higher ventricular eccentricity index, particularly during exercise, suggesting that EAT exerted a mechanical restraining effect on the pericardium [48]. The global longitudinal strain (GLS), which measures the rate of myocardial length change, can be used as an indicator of diastolic dysfunction even in the presence of normal LVEF levels [49]. Furthermore, there exists an independent association between increased EAT volume and impaired GLS [50]. In HFpEF patients, EAT plays a crucial role in determining left ventricular (LV) GLS and is not affected by BMI [51]. Worse GLS and higher right filling pressures may be related to the effect of the asymmetric distribution of EAT around the heart [52, 53]. Additionally, elevated EAT is positively associated with increased right ventricular end-diastolic pressure and pulmonary vascular resistance, resulting in reduced exercise capacity among patients with HFpEF [52].

### 4.2 Exercise Reserve Capacity

Cardiopulmonary reserve is significantly reduced in HFpEF patients during exercise. In comparison to individuals without HF, HFpEF patients have no significant difference in oxygen demand during exercise, but they experience a substantial decline in myocardial oxygen supply [54]. Analysis of clinical data revealed that the regional accumulation of fat in HFpEF patients, particularly the accumulation of EAT surrounding the heart, has a more pronounced influence on their exercise cardiopulmonary reserve [48]. The 6-minute walk distance (6MWD) is a useful measure for evaluating the exercise tolerance of individuals with chronic HF. After adjusting for age, sex, and BMI, it has been observed that the accumulation of EAT is associated with a decrease in 6MWD, indicating a detrimental effect on exercise tolerance [55]. In addition, the assessment of exercise reserve capacity can be achieved through the measurement of peak oxygen consumption (VO_2_) during cardiopulmonary exercise testing, which serves as an objective indicator for prognosis evaluation. In the case of HFpEF, EAT accumulation exhibits a negative correlation with VO_2_, and this correlation persists even after adjusting for BMI or waist circumference [35, 52]. However, the findings of Haykowsky *et al*. [56] were inconsistent with previous research, as they discovered that elderly obese patients (age ≥60 years; BMI ≥30 kg/m^2^) with HFpEF had significantly lower EAT volume compared to the healthy control group. Additionally, they observed a positive correlation between EAT and both VO_2_ and 6MWD. This discrepancy may be attributed to three potential factors: (1) Variations in measurement techniques: EAT can be assessed using MRI or echocardiography in clinical and research settings, and discrepancies may arise due to the utilization of different ways; (2) Measurement criteria: Currently, there is a lack of uniformity in the criteria used to measure EAT, resulting in inconsistent locations and dimensions of assessment across studies; (3) Population selection: HFpEF is a clinical syndrome, and the study conducted by Haykowsky *et al*. [56] primarily focused on obese elderly with HFpEF. It is important to note that the characteristics of the selected populations may have an impact on the obtained results.

### 4.3 Inflammation

The role of inflammation in HFpEF has garnered increased attention particularly in recent years. In 2020, according to the results of proteomic evaluation from the “PROMIS-HFpEF” study, Sanders-van *et al*. [57] proposed the comorbidity-inflammation paradigm to summarize the development of HFpEF with one major pathophysiological mechanism as much as possible, but the concept of “comorbidity” involved multiple diseases and contains multiple mechanisms. As a VAT, EAT is rich in immune-related cells such as macrophages and lymphocytes in addition to adipocytes. Its volume is independently associated with serum C-reactive protein levels [34]. The chronic inflammatory state observed in EAT may be attributed to the following: (1) The activation of inflammatory signaling pathways, such as the nuclear factor kappa-B (NF-κB) pathway and c-Jun N-terminal kinase (JNK) pathway. (2) Autophagy and pyroptosis pathways are also activated in EAT. Burgeiro *et al*. [58] discovered increased expression levels of autophagy-related genes and proteins in the EAT of HF patients. The activation of the inflammasome-mediated pyroptosis pathway was observed in the EAT of HFpEF mice [59]. (3) EAT showed higher production of mitochondrial reactive oxygen species (ROS) compared to subcutaneous adipose tissue (SAT), and proteomic analysis revealed significant variations in protein expression within the oxidative stress pathway [60]. Decreased expression of the thermogenic protein UCP-1 in damaged EAT triggers the production of ROS [61]. This chronic inflammatory state can have profound impacts on adjacent myocardium or blood vessels through various mechanisms: (1) EAT secretes multiple inflammatory factors, including tumor necrosis factor-α (TNF-α), interleukin-1 (IL-1), and interleukin-6 (IL-6). Compared to the control population, the expression of pro-inflammatory factors in EAT was significantly elevated in CAD, T2DM, and HFpEF patients [62, 63]. (2) The expression of anti-inflammatory factors such as adiponectin in EAT are significantly diminished [64]. (3) EAT can release chemotactic proteins such as monocyte chemotactic protein-1 (MCP-1), which may attract inflammatory cells like macrophages [65]. The expression of hypoxia-inducible factor-1α (HIF-1α) is significantly increased in the adipose tissue, particularly in EAT, of HFpEF patients with obesity, leading to the recruitment of macrophages and the promotion of M1-type polarization [66]. Several observational studies have demonstrated that the administration of statins in HFpEF patients substantially reduces the risk of mortality [67]. Statins have been shown to decrease EAT metabolic activity and reduce the secretion of inflammatory mediators by targeting non-lipid-lowering effects such as inflammation [68, 69]. Proprotein convertase subtilisin/kexin type-9 inhibitors (PCSK9i) inhibitors showed a more effective reduction in EAT, which was independent of low-density lipoprotein-C [70]. Sodium-glucose cotransporter 2 inhibitors (SGLT2i) could reduce the secretion of IL-6 and other inflammatory factors by inhibiting the production of EAT [71]. Whether anti-hyperlipidemic drugs or anti-hyperglycemic drugs reduce the metabolic or cardiovascular risk of HFpEF by modulating the inflammatory response in EAT remains to be determined by further clinical studies.

### 4.4 Myocardial Structure and Function

Ventricular fibrosis and hypertrophy are significant pathological alterations in ventricular remodeling, serving as the foundation for HFpEF development and a crucial manifestation of cardiac decompensation [25]. Although fibrosis is a significant pathological manifestation of HF, the association between EAT and myocardial fibrosis is limited to HFpEF [36]. Previous prospective studies employing endomyocardial biopsies demonstrated that 93% of HFpEF patients exhibited myocardial fibrosis and 88% exhibited cardiomyocyte hypertrophy [25]. EAT is located near the heart and has a significant impact on ventricular remodeling through various mechanisms. Wu *et al*. [32] used cardiac MRI to measure extracellular volume (ECV) as a quantitative measure of myocardial fibrosis. The findings of this study demonstrated a significant association between EAT and ECV regardless of age, T2DM, hypertension, LVEF, and other confounding factors. These results imply that EAT independently contributes to the development of myocardial fibrosis.

Previous studies have demonstrated that EAT can secrete mediators like connective tissue growth factor and transforming growth factor-β (TGF-β) involved in fibrosis remodeling directly. Additionally, the secreted factors originating from EAT derived from human have been observed to initiate fibrosis in rat atrial models directly [72]. EAT secretes IL-6, TNF-α and other inflammatory factors, playing local toxic effects. This chronic inflammatory effect may cause pathological changes directly, including fibrosis and hypertrophy of the surrounding myocardium [67]. In addition, EAT can also produce pro-inflammatory and pro-fibrotic mediators through the infiltrated pro-inflammatory macrophages, so that fibroblasts can differentiate into myofibroblasts and secrete more pro-fibrotic mediators [73]. Hao *et al*. [74] clarified the role of EAT-microRNA (miRNA) axis in adverse myocardial remodeling. *In vitro* experiments demonstrated that EAT affects the levels of ROS in cardiomyocytes through secreting miRNA, resulting in cardiomyocyte hypertrophy and activation of cardiac fibroblasts. Agra *et al*. [75] found that after isoproterenol stimulation, the expression of *p53* gene was only increased in EAT tissue, but not in SAT tissue. This suggests that the overactivation of the sympathetic nervous system may mediate the overexpression of the *p53* gene in EAT tissue in HF patients. Genomic analysis showed that *p53* gene was specifically activated in the late stage of hypertrophic myocardium, indicating that EAT may play an important role in the development of HF through p53 [76].

### 4.5 Arrhythmia 

Arrhythmia is a prevalent complication in patients with HFpEF [15]. A comprehensive meta-analysis of 61 studies revealed a heightened incidence of AF in HFpEF patients [77]. The presence of AF exacerbates the strain on the left atrium, resulting in diminished functionality, and further promotes the development and progression of arrhythmias in HFpEF patients [15]. Utilizing cardiac MRI, it was observed that HFpEF patients with AF had greater accumulation of EAT surrounding the atrium than those without AF, while it was no significant difference in EAT around the ventricle [78]. The primary cause of AF is atrial myocardial fibrosis [79]. EAT contributes to the myocardial dysfunction by exacerbation of inflammation and cardiac fibrosis, and ultimately leads to prolonged and depolarization of cardiomyocyte action potential [80, 81]. Consequently, this process promotes the occurrence of arrhythmias and the progression of HF. Treatment with EAT conditioned medium enhanced the population of myofibroblasts, elevating the expression of fibrosis markers like collagen and TGF-β and inducing atrial fibrosis [82].

Furthermore, EAT explants conditioned medium derived from AF patients could impede electrical conduction and enhance conduction heterogeneity in neonatal rat ventricular myocytes [83]. Lin *et al*. [84] first confirmed that the direct interaction between EAT and the atrium could trigger abrupt discharges through the induction calcium overload in the left atrium. This phenomenon may be attributed to the release of free FAs, adipokines, or neurohumoral factors from EAT. When the number of FAs released by fat cells in EAT exceeds the oxidative capacity of the mitochondria, it will occur lipid toxic effect. These effects subsequently lead to the endoplasmic reticulum (ER) dysfunction, dysregulated calcium levels, and increased ROS production in cardiomyocytes [81]. The resulting overload of cytoplasmic calcium in the cardiomyocytes, along with the spontaneous release of calcium, leads to the prolongation of action potentials and the occurrence of early and delayed post-depolarization. Studies have found that stearic acid has the function of FAs, which can interfere the T-tube structure of atrial myocytes and modify the ionic current of cardiomyocyte membranes, thereby leading to the occurrence of arrhythmias [85]. EAT can also disrupt the intracellular and extracellular transmission of calcium and potassium ions through inflammatory factors or adipokines, leading to the abnormal electrical activity [86]. In addition, adrenergic stimulation has been shown to enhance the entry, storage, and release of calcium ions in the myocardium, facilitating the initiation of calcium-dependent electrical activity and leading to the development of atrial tachyarrhythmias [87]. It is worth noting that EAT contains a large amount of adrenergic and cholinergic nerves, which can affect the autonomic nervous function of myocardium [88]. EAT can induce the activation of the central sympathetic nervous system (SNS) through releasing epinephrine and norepinephrine [88]. In patients with HF, EAT serves as a significant source of norepinephrine, exhibiting NE levels that are 5.6-fold higher in peripheral fat and 2-fold higher in plasma [89]. Consequently, the excessive activity of SNS mediated by EAT contributes to the development of arrhythmia, representing one of the crucial mechanisms of HF. SGLT2i have demonstrated the potential to reduce left atrial enlargement in patients with HFpEF and to mitigate the incidence and intensity of spontaneous calcium release events in left atrial cardiomyocytes, thereby reducing the frequency of arrhythmias [90]. P-wave dispersion is an important indicator for predicting atrial arrhythmias and is independently correlated with EAT volume. Dapagliflozin as an SGLT2i, can change P-wave dispersion by reducing EAT volume [91]. However, the precise mechanism by which SGLT2i treats arrhythmias through EAT requires further investigation.

### 4.6 Metabolism

Substantial changes in cardiac metabolism and energy utilization occur in HF states compared to normal physiological states. Notably, there is a marked reduction in FAs metabolism within the failing heart. Therefore, the failing heart must intensify its reliance on alternative substrates, including glucose, ketone bodies, and amino acids to adequately meet its energy requirements [92]. In contrast to patients with HFrEF, those with HFpEF exhibited a higher prevalence of obesity and T2DM. However, the analysis of myocardial metabolites revealed diminished levels of FAs oxidation [93]. Furthermore, HFpEF patients were observed to exhibit low levels of tricarboxylic and ketone metabolism, indicating reduced utilization of alternative energy sources. In the Aldosterone Antagonist Therapy Heart Failure Trial with Preserved Cardiac Function (TOPCAT), HFpEF patients with comorbidities such as T2DM, obesity, and advanced symptoms had the most unfavorable prognosis [94]. Regardless of the presence of metabolic syndrome, the myocardial structure of HFpEF patients displayed significant alterations. However, individuals with comorbidities demonstrated more pronounced myocardial remodeling [95], implying the crucial involvement of metabolic abnormalities in the progression of HFpEF.

In a prospective study, it was observed that the density of EAT serves as a predictive factor for cardiometabolic risk in patients with HFpEF [96]. Through proteomic analysis, it was determined that there were significant differences in the expression of proteins related to lipid metabolism, inflammation, and mitochondrial dysfunction between HFpEF and non-HFpEF patients [97, 98]. Furthermore, it was found that the expression of genes involved in lipid storage and lipolysis in EAT is notably reduced in HF patients, leading to an increase in the level of free FAs, which aggravates the toxic effect of lipids on the heart [98]. Due to the BAT characteristics of EAT, there is a notable upregulation of peroxisome proliferator-activated receptor-α (PPAR-α) within it, which plays a crucial role in modulating the expression of various target genes associated with FAs metabolism, FAs oxidation, and glucose metabolism [99]. This leads to an enhanced absorption, utilization, and catabolism of FAs in tissues. In individuals with HF, EAT exhibits impaired glucose and lipid metabolism, accompanied by a significant reduction in PPAR-α levels [99], which affects the energy supply of the heart. The EAT surrounding the heart in patients with HF exhibits a significant reduction in beneficial fatty acids, especially docosahexaenoic acid (DHA) [99]. DHA plays a crucial role in eliminating ROS, delaying the opening of the mitochondrial permeability transition pore, and enhancing mitochondrial antioxidant capacity.

Diabetes-associated HFpEF is considered to be a specific phenotype of diabetic cardiomyopathy [100]. T2DM has an independent effect on cardiopulmonary insufficiency, as shown by a decrease in VO_2_ peak. Although BMI has been found to influence VO_2_ peak independently of T2DM, it is not clear whether EAT independently impacts VO_2_ peak [101]. Another study found that in T2DM patients, EAT was positively associated with the combined endpoint of cardiovascular events and mortality [102]. These two studies suggest that EAT may play a promoting role in the development and progression of HFpEF by adjusting myocardial metabolic factors. Several anti-hypoglycemic drugs have been shown to reduce EAT volume [103]. In T2DM patients, EAT was negatively correlated with LV diastolic function and insulin sensitivity [104]. Pioglitazone exerts insulin sensitization effect by activating PPAR-γ. PPAR-γ is highly expressed in adipose tissue, and its expression is reduced when insulin resistance occurs. Pioglitazone may increase insulin sensitivity by decreasing EAT volume and reducing pro-inflammatory gene expression in EAT [105, 106]. Pioglitazone can also improve left ventricular diastolic function by reducing EAT volume [104]. SGLT2i and glucagon-like peptide-1 receptor agonists (GLP-1RA) have been shown to have cardioprotective effects independent of hypoglycemia [107]. Treatment of EAT explants with dapagliflozin can reduce the secretion of inflammatory factors and improve the renewal of mature and functional adipocytes with protecting the insulin response [108]. GLP-1RA may be more effective in reducing EAT compared to SGLT2i [103], however the mechanism of GLP-1RA needs to be further explored.

### 4.7 Microvascular Function

In a cohort study of 106 HFpEF patients, of those without obstructive CAD, 81% had coronary microvascular dysfunction (CMD) [109]. CMD plays a role in the pathophysiology of HFpEF formation [110]. Due to their small diameter, microvessels are sensitive to hemodynamic changes and can directly impact myocardial perfusion. The contraction or dilation of microvessels leads to changes in blood flow resistance, subsequently affecting the oxygen supply to the myocardium [111]. When microvascular dysfunction occurs, the reactive hyperemic capacity of cardiomyocytes decreases significantly [110]. The EAT volume of postmenopausal women with T2DM was significantly higher than those without T2DM, and had harmful effects on myocardial microvascular function [112], indicating that metabolism-related factors play a unique role on microvascular function in the female population. EAT has potential to function as a biomarker for the early detection of microvascular dysfunction. For patients with normal myocardial perfusion, the volume of EAT can be used to predict the occurrence of absolute myocardial blood flow congestion and decrease myocardial perfusion reserve [113]. Furthermore, EAT is associated with impaired coronary flow reserve (CFR) [114]. CFR refers to the capacity of a coronary artery to supply additional blood flow under specific loads. Even in the absence of coronary artery disease, elevated volume of EAT is associated with an increased risk of angina and other adverse cardiovascular outcomes [115]. The effect of EAT on CMD may not be related to microvascular endothelial function [116]. As a type of BAT, EAT possesses higher thermogenic capacity and contains more mitochondria than white fat. When the browning of EAT is impaired, the expression levels of thermogenic protein UCP-1 and PPAR-α decrease, leading to impaired mitochondrial function in EAT. This impairment results in increasing ROS and other inflammatory factors, ultimately leading to microvascular dysfunction in the peripheral myocardial tissue [61, 117].

## 5. Conclusions

At present, the mechanism and treatment of HFpEF are constantly improving. As one of the most important VAT, EAT is attached to the surface of heart. EAT can influence cardiac metabolism and function through paracrine effects in addition to exerting mechanical effects. This review focuses on EAT and provides an in-depth summary of EAT potential mechanisms that may influence HFpEF. In recent years, through the continuous understanding of EAT, we have fully discussed the effects of EAT on myocardial structure, energy application, electrical activity, and microvessels around the myocardium by regulating inflammatory, metabolic, and neurological factors. The purpose of this review is to improve our understanding and management of HFpEF. Future studies should continue to investigate the mechanism between EAT and HFpEF, and explore interventions targeting EAT to improve the prognosis of HFpEF patients.

## Abbreviations

AF, Atrial fibrillation; BAT, Brown adipose tissue; BMI, Body mass index; CAD, Coronary artery disease; CFR, Coronary flow reserve; CMD, Coronary microvascular dysfunction; DHA, Docosahexaenoic acid; EAT, Epicardial adipose tissue; ECV, Extracellular volume; FAs, Fatty acids; GLP-1RA, Glucagon-like peptide-1 receptor agonists; GLS, Global longitudinal strain; HF, Heart failure; HFmrEF, Heart failure with mid-range ejection fraction; HFpEF, Heart failure with preserved ejection fraction; HFrEF, Heart failure with reduced ejection fraction; IL-1, Interleukin-1; IL-6, Interleukin-6; LVEF, Left ventricular ejection fraction; MRI, Magnetic resonance imaging; PPAR-α, Peroxisome proliferator-activated receptor-α; ROS, Reactive oxygen species; SAT, Subcutaneous adipose tissue; SGLT2i, Sodium-glucose cotransporter 2 inhibitor; SNS, Sympathetic nervous system; TGF-β, Transforming growth factor-β; TNF-α, Tumor necrosis factor-α; UCP-1, Uncoupling protein-1; VAT, Visceral adipose tissue; VO_2_, Peak oxygen consumption; 6MWD, 6-minute walk distance.
